# Functional Stratification and Postoperative Outcomes in Endobronchial Tumor Surgery: A Dedicated Center Experience

**DOI:** 10.3390/jcm15145605

**Published:** 2026-07-17

**Authors:** Radu Matache, Silviu Gabriel Vlăsceanu, Beatrice Mahler, Alexandru Stoichiță, Camelia Alexandra Paruschi, Alina Elena Tucana, Irina Niță, Andrei Cristian Bobocea, Cornel Florentin Savu

**Affiliations:** 1 Department of Thoracic Surgery, “Marius Nasta” National Institute of Pneumology, 050150 Bucharest, Romania; radu.matache@gmail.com (R.M.); silviu.vlasceanu@drd.umfcd.ro (S.G.V.); camelia-alexandra.paruschi@rez.umfcd.ro (C.A.P.); alina-elena.tucana0226@rez.umfcd.ro (A.E.T.); andrei.bobocea@gmail.com (A.C.B.); drsavu25@yahoo.com (C.F.S.); 2 Thoracic Surgery I, University of Medicine and Pharmacy “Carol Davila”, 020021 Bucharest, Romania; 3 Department of Pneumology, “Marius Nasta” National Institute of Pneumology, 050150 Bucharest, Romania; 4 Pneumoftisiology II Discipline, Faculty of Medicine, University of Medicine and Pharmacy “Carol Davila”, 020021 Bucharest, Romania; 5 Department of Oncology, Medicover Hospital, 020331 Bucharest, Romania; irina.nita0910@gmail.com

**Keywords:** endobronchial tumor, spirometry, diffusing capacity for carbon monoxide (DLCO), lung resection, postoperative complications, preoperative cardio-respiratory evaluation

## Abstract

**Background**: Endobronchial tumors represent a heterogeneous spectrum of benign and malignant histopathological types. Selecting the optimal surgical strategy remains a multidisciplinary challenge. This study evaluates how the integration of spirometry, DLCO, and CPET can objectively stratify cardiorespiratory risk and guide surgical decision-making regarding resection type, postoperative complications, and perioperative mortality. **Methods**: A single-center, retrospective study was conducted between 2020 and 2025, evaluating an initial screening cohort of 93 patients with endobronchial masses. Following multidisciplinary tumor board review based on functional and anatomical criteria, 36 patients were excluded from major resection due to extensive disease or prohibitive functional risk, while 57 underwent tailored surgical interventions, including sleeve resections, lobectomies, bilobectomies, and pneumonectomies. Preoperative evaluation included spirometry, DLCO, cardiological assessment, CT, and autofluorescence bronchoscopy (AFB). **Results**: Spearman correlation analysis demonstrated that advanced age and higher ASA scores significantly correlated with reduced preoperative DLCO (*p* < 0.0001). The overall postoperative morbidity rate was 36.84% (*n* = 21) and perioperative mortality was 5.26% (*n* = 3). Stratified analysis by surgical magnitude demonstrated a significant escalation in overall complication rates from parenchymal-sparing sleeve resections (17.6%, 95% CI: 3.8% to 43.4%) to standard lobectomies (40.7%, 95% CI: 22.4% to 61.2%), bilobectomies (50.0%, 95% CI: 15.7% to 84.3%), and radical pneumonectomies (60.0%, 95% CI: 14.7% to 94.7%; *p* = 0.042). Severe morbidity (Clavien–Dindo Grade III–IV) was significantly lower in the sleeve resection subgroup (5.8%) compared to pneumonectomies (40.0%, *p* = 0.015). Fatal outcomes occurred exclusively following major resections and were attributable to ARDS and acute cardiovascular events. **Conclusions**: A multi-parametric preoperative protocol provides an informative framework for risk stratification in endobronchial tumor surgery, helping to describe baseline reserves and explore post-resection trends. However, the anatomical and hemodynamic magnitude of radical pneumonectomy constitutes an independent risk modifier that cannot be fully captured by preoperative functional testing alone, highlighting the exploratory nature of these single-center retrospective observations rather than a definitive algorithmic validation.

## 1. Introduction

Lung cancer remains the leading cause of oncology-related mortality worldwide and is the second most prevalent malignancy in both men and women [1]. Within this broad oncological landscape, primary endobronchial tumors represent a distinct and relatively rare clinical entity, encompassing a highly heterogeneous histopathological spectrum that includes both benign lesions and aggressive malignancies [2]. The comprehensive management of patients presenting with endobronchial masses necessitates a rigorous, multi-stage diagnostic approach focused on accurate histopathological characterization, precise localization of the primary tumor, evaluation of regional lymph node involvement, and systematic staging for distant metastases [3].

Beyond oncological staging, the preoperative optimization of these patients must strictly adhere to established cardiopulmonary functional protocols for lung resection [4]. This includes the utilization of validated risk stratification models, such as the Thoracic Revised Cardiac Risk Index (ThRCRI), to identify and mitigate perioperative cardiovascular hazards [5].

Historically, centrally located endobronchial neoplasms routinely necessitated radical parenchymal resections, with pneumonectomy long serving as the unavoidable surgical standard to achieve complete oncological margin clearance [4]. However, preserving functional lung tissue is paramount for maintaining optimal long-term respiratory capacity and a higher postoperative quality of life. Consequently, parenchymal-sparing approaches—specifically bronchial sleeve resections, whether performed in isolation or combined with lobectomy or bilobectomy—have emerged as highly favorable alternatives to pneumonectomy for central lesions [3]. Nevertheless, the critical challenge does not reside in the surgical technique itself, but in the rigorous preoperative identification of candidates for whom such parenchymal-sparing strategies are both oncologically appropriate and functionally safe. Even within a structured institutional algorithm, predictive limitations persist, as demonstrated by the postoperative outcomes reported herein.

Implementing these sophisticated lung-sparing techniques relies on a comprehensive anatomical and functional triage to optimize candidate selection. While an integrated multi-system diagnostic approach combining high-resolution computed tomography (CT), flexible autofluorescence bronchoscopy (AFB), standard spirometry, and diffusing capacity of the lung for carbon monoxide (DLCO) provides an informative staging framework, its application must be tailored to the clinical presentation. In clinical practice, patients presenting with large, highly obstructive endobronchial masses or acute secondary atelectasis frequently require immediate surgical intervention as a primary diagnostic and therapeutic measure, bypassing extensive pre-resection endoscopic mapping. Furthermore, while advanced physiological optimization is critical, cardiopulmonary exercise testing (CPET) is not a uniform mandate for every surgical candidate; rather, it is selectively reserved for stratifying individuals with borderline resting functional reserves.

Clinically, endobronchial tumors frequently masquerade as benign respiratory conditions due to non-specific symptoms such as persistent cough, hemoptysis, recurrent obstructive pneumonia, localized wheezing, and chest pain [2]. Radiological evaluation via volumetric CT is critical, as it identifies key diagnostic markers such as the bronchus sign [6], the bronchial cutoff sign [7], and intratumoral air bronchograms. These findings, when analyzed according to the Tsuboi classification [8], provide essential data regarding endobronchial tumor diameter, transmural penetration, and proximity to the hilum.

This radiological workup is complemented by flexible bronchoscopy enhanced with autofluorescence imaging, which represents a mandatory institutional protocol for all patients undergoing lung resection, as mucosal evaluation can directly dictate the surgical strategy; even in urgent scenarios where surgery serves as the primary diagnostic and therapeutic measure, standard bronchoscopy is systematically performed prior to resection to assess macroscopic margins and potential carina involvement. Within this framework, the addition of AFB serves as a valuable extra tool for mapping subtle mucosal alterations across the oncogenic spectrum, from low-grade to high-grade dysplasia and invasive carcinoma [9]. This advanced modality allows for the detection of subtle sub-visual irregularities, including mucosal thickening or nodular patterns [10], meaning that while standard endoscopic evaluation is routine, the advanced AFB component itself is not an absolute, rigid prerequisite prior to selecting the procedure in every case.

Despite the proven advantages of parenchymal preservation, a standardized, universally accepted pre-operative functional protocol that effectively balances oncological safety against post-resection mortality and morbidity remains a subject of ongoing debate. While standard guidelines offer general frameworks, a single-center validation of how integrated functional and anatomical scoring predicts the exact scale of resection—and guards against severe post-operative complications like Acute Respiratory Distress Syndrome (ARDS) or acute cardiovascular events—is highly valuable.

In the context of evaluating candidates for endobronchial tumor surgery, accounting for local respiratory comorbidities is essential. Pre-existing parenchymal damage and chronic obstructive conditions significantly compromise baseline ventilatory function, thereby reducing the functional reserve required to tolerate major lung resections. Recent multi-center data from the Romanian population highlights that cumulative structural lung changes can severely accelerate the severity of ventilatory impairment and obstructive disease onset [11]. Therefore, a rigorous triparametric functional assessment becomes even more critical in surgical cohorts where baseline respiratory parameters are already degraded by overlapping chronic pathologies.

Synthesizing current literature alongside the latest NCCN and ESMO guidelines, this study aims to describe our institutional experience regarding preoperative risk stratification in a cohort of 93 screened patients, focusing on the exploratory assessment of associations between functional testing, surgical selection, and postoperative outcomes.

## 2. Materials and Methods

### 2.1. Study Design and Patient Selection

**Figure 1:**
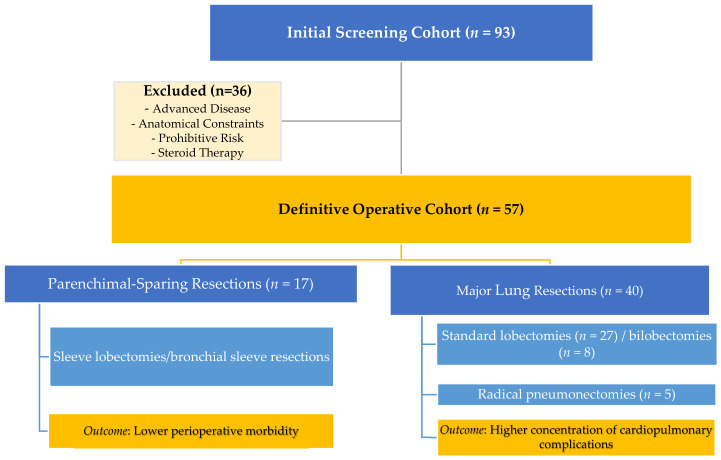
Flowchart of the patient screening, exclusion criteria, and definitive surgical treatment allocation workflow.

A single-center, retrospective cohort study was conducted between 2020 and 2025 to evaluate an integrated preoperative selection algorithm for patients presenting with endobronchial masses. The initial screening pool comprised 93 consecutive patients presenting with clinical and radiological evidence of lung neoplasms. Following a multi-system diagnostic and functional triage, 36 patients were excluded from surgical resection, yielding a final operative cohort of 57 patients who underwent tailored surgical treatment based on their baseline functional reserves and localized anatomical criteria, as illustrated in the patient selection flowchart (Figure 1). Interventions encompassed both parenchymal-sparing strategies (bronchial sleeve resections) and major radical lung resections (lobectomies, bilobectomies, and pneumonectomies).

To manage cases with overlapping clinical challenges, a strict hierarchical decision-making pathway was applied. The primary criteria for exclusion were prioritized based on immediate functional safety and oncological resectability. The 36 exclusions were classified under four mutually exclusive primary reasons:

- Severe functional respiratory compromise (*n* = 14): Patients presenting with a predicted postoperative forced expiratory volume in 1 s (ppoFEV_1_) or predicted postoperative DLCO below 40%, who failed to demonstrate adequate metabolic compensation during advanced cardiopulmonary exercise testing, achieving a peak oxygen consumption (VO_2_peak) of less than 10 mL/kg/min.
- Advanced or unresectable oncological stage (*n* = 12): Patients found during baseline staging to have extensive mediastinal structures invasion (T4) or distant organ metastasis (M1), rendering surgical resection non-beneficial.
- High-risk cardiovascular comorbidities (*n* = 6): Patients classified as ASA IV or presenting with an elevated ThRCRI, including recent myocardial infarction within 3 months, decompensated New York Heart Association (NYHA) class III/IV heart failure, or uncontrolled malignant arrhythmias.
- Documented patient refusal (*n* = 4): Patients who met all anatomical and functional criteria for major resection but opted for alternative oncological treatments or palliative care pathways.
- In instances where patients exhibited concurrent respiratory and cardiovascular limitations, the primary cause for exclusion was allocated to the organ system posing the most immediate threat to perioperative survival. A detailed comparative analysis of the clinical, functional, and oncological profiles of both the excluded and operated cohorts is provided in Supplementary Table S1.

This study was performed in strict accordance with the ethical principles of the Declaration of Helsinki. The study design was initiated and received formal approval from the Institutional Research Ethics Committee of the “Marius Nasta” Institute of Pneumophthisiology (Approval No. 12707/31 May 2025) in May 2025, at which point retrospective data collection from institutional archives commenced. Clinical data from patients treated between January 2020 and the study initiation date were retrieved retrospectively from pre-existing electronic medical records, with no research-related intervention or prospective patient enrollment performed prior to ethics approval. Upon hospital admission, all patients had provided written informed consent allowing the pseudonymized use of their clinical, imaging, histopathological, and functional data for scientific research purposes.

### 2.2. Inclusion Criteria

Patients were included in the definitive operative cohort if they met all of the following integrated criteria: (1) definitive radiological and endoscopic confirmation of a central or peripheral endobronchial mass located within the main, intermediate, or lobar bronchi via CT and flexible autofluorescence bronchoscopy mapping; (2) localized oncological disease demonstrating anatomical resectability without extensive mediastinal invasion or distant metastases; (3) adequate cardiorespiratory reserve defined as a baseline forced expiratory volume in 1 s (FEV_1_) and a DLCO both greater than 40% of the predicted values, supplemented by CPET; and (4) staging categorized as Stage II (N1) or Stage IIIA (N2) disease, according to the ninth edition of the TNM classification [12]. Although neoadjuvant systemic therapy was considered for documented N2 cases as per NCCN and ESMO guidelines [13,14], all patients in this cohort proceeded directly to primary surgical resection following multidisciplinary tumor board review. Preoperative mediastinal evaluation relied on chest computed tomography and positron emission tomography scans, with invasive mediastinal staging via endobronchial ultrasound or mediastinoscopy reserved for patients with ambiguous lymph node enlargement rather than performed systematically. Direct surgical resection in clinical stage IIIA (N2) disease was restricted to single-station N2 involvement (N2a under the TNM 9 framework) deemed technically resectable at multidisciplinary review, or to unexpected N2 disease discovered during postoperative histopathological examination; single-station versus multistation N2 disease was strictly defined according to the 9th edition guidelines. Adjuvant chemotherapy, immunotherapy, or radiotherapy was systematically proposed postoperatively for all eligible patients with confirmed N2 disease.

### 2.3. Exclusion Criteria

Exclusion criteria for major surgical resection were defined based on the following objective criteria: (1) advanced disseminated malignancy presenting multi-station N2/N3 lymph node involvement or extensive mediastinal invasion unsuited for primary surgical clearance; (2) anatomical constraints of the proximal airway, including tumors originating within or extensively infiltrating the mid-to-upper trachea, tracheobronchial junction, or main carina where standard reconstructive techniques were unfeasible; (3) prohibitive functional cardiorespiratory risk under general anesthesia with single-lung ventilation (including baseline FEV_1_ or DLCO below 40% of predicted values without satisfactory CPET stratification); and (4) ongoing long-term systemic corticosteroid therapy due to its documented adverse impact on bronchial anastomotic healing.

### 2.4. Data Collection and Variables

A meticulous retrospective review of institutional electronic medical records was performed to extract baseline clinical, demographic, and paraclinical parameters. The consolidated database encompassed patient demographics (chronological age, biological sex, and American Society of Anesthesiologists [ASA] physical status score) alongside presenting respiratory and systemic symptoms (cough, hemoptysis, obstructive pneumonia, wheezing, and chest pain). Preoperative cardiorespiratory metrics detailed baseline spirometric values (FEV_1_, FVC, and the FEV_1_/FVC ratio), absolute and age-predicted DLCO variances, and advanced CPET parameters. Laboratory profiles tracked complete blood counts, serum biochemistry, and blood-derived tumor markers, specifically Cyfra 21-1, CEA, SCC-Ag, CgA, NSE, and urinary 5-HIAA. Anatomical, endoscopic, histopathological, and surgical variables (detailed in Section 2.6, Section 2.7 and Section 2.8) were similarly extracted and cross-verified by the institutional multidisciplinary tumor board.

### 2.5. Statistical Analysis

Statistical analysis was performed using MedCalc statistical software (Version 22.0, MedCalc Software Ltd., Ostend, Belgium). The distribution of continuous variables was systematically evaluated for normality using the D’Agostino-Pearson test. Continuous variables with a normal distribution are expressed as means plus or minus standard deviation (SD). Continuous variables with a non-normal distribution or small subgroup sample sizes are expressed as medians with interquartile ranges (IQR) and were compared using the non-parametric Kruskal–Wallis test. Categorical variables are expressed as absolute frequencies and percentages (*n*, %).

To directly address the primary clinical questions regarding postoperative risk stratification while accounting for the small number of adverse events and the limited cohort size (*n* = 57), conventional multivariable regression models were strictly avoided to prevent statistical overfitting and overinterpretation. Instead, direct comparisons of postoperative complication rates, severity grades, and mortality across the four surgical resection types and across the three distinct preoperative testing pathways were performed using Fisher’s exact test or the exact Chi-squared test for multi-group tables. Furthermore, exact binomial confidence intervals (95% CI) were computed for all subgroup complication and mortality rates to provide an accurate reflection of statistical uncertainty. Spearman rank correlation coefficients were restricted solely to describing baseline physiological interactions between pre-resection functional parameters and demographic variables, rather than serving as a predictive framework for clinical outcomes. Data significance was tested against a standard two-tailed alpha threshold, where an exact *p*-value of less than 0.05 was considered statistically significant.

### 2.6. Computed Tomography Evaluation

**Figure 2:**
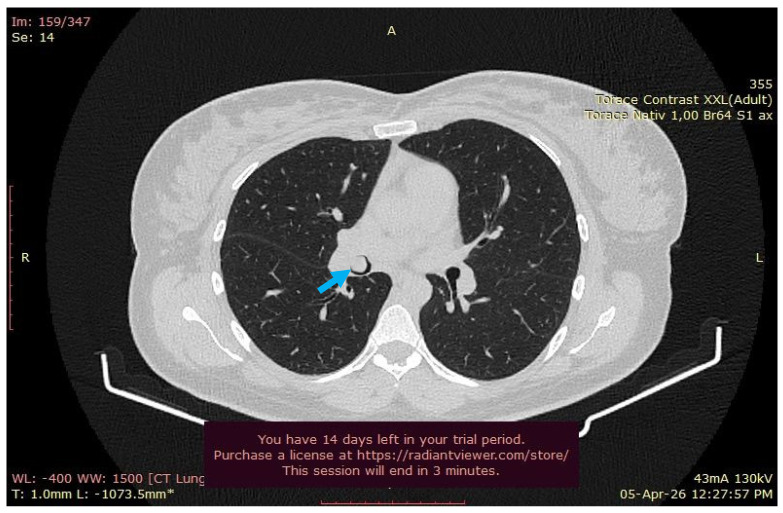
Contrast-enhanced axial chest CT (lung window) demonstrating a large endoluminal endobronchial mass occupying the right main bronchus (blue arrow), with associated bronchial cutoff sign. (Personal archive).

High-resolution thoracic CT was used to assess tumor dimensions, airway involvement (categorized per the Tsuboi classification), and resectability-relevant radiographic signs (Figure 2); full acquisition and classification details are provided in Supplementary Material, Section S1.

### 2.7. Flexible Autofluorescence Bronchoscopy and Airway Mapping

**Figure 3:**
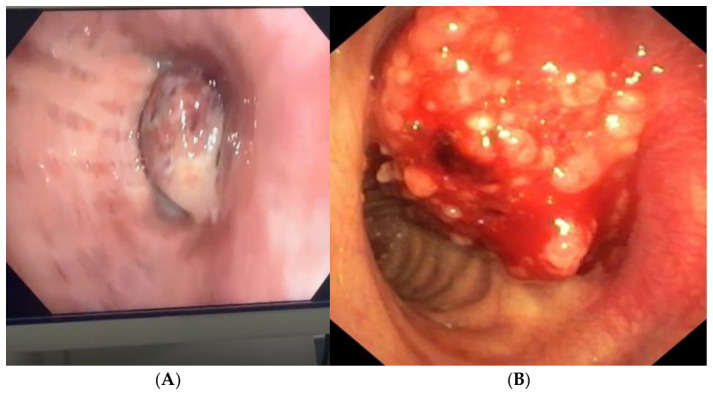
(**A**,**B**) Flexible white-light bronchoscopy findings in two representative cases of endobronchial malignancy. (**A**) A sessile, broad-based endoluminal mass with a smooth, glistening surface causing partial luminal occlusion of the affected bronchus, with intact surrounding mucosa. (**B**) A large, polypoid endobronchial mass with a nodular, friable surface and active contact-induced hemorrhage, producing near-complete bronchial luminal obstruction; cartilaginous rings remain partially visible at the inferior margin. (Personal archive).

Flexible video-bronchoscopy was performed under local topical anesthesia using high-definition systems equipped with autofluorescence imaging (AFI) capabilities. An initial step involved the real-time endoscopic verification of the tracheobronchial tree to correlate macro-anatomical features with preoperative CT datasets. During this evaluation, a comprehensive characterization of each endobronchial tumor was recorded, including its precise localization, macroscopic size, structural configuration, and implantation base (pedunculated or broad-based sessile). Furthermore, the analysis tracked the appearance of the peritumoral mucosa under both white-light and autofluorescence modes, the presence of adjacent necrotic debris or fibrin deposits, and the quantification of luminal bronchial occlusion (Figure 3).

The baseline mucosal vascular patterns, tissue friability, and propensity for contact-induced hemorrhage upon instrumentation were also systematically assessed. Subsequently, distal bronchial secretions were collected for cytological and microbiological screening, alongside multiple serial forceps biopsies from the lesion core and margins to establish the definitive histopathological profile. The mucosal boundary assessment was conducted with the operating thoracic surgeon present, enabling real-time identification of macroscopically healthy, disease-free bronchial cartilage rings proximal to the tumor —serving as an anatomical determinant for establishing the technical feasibility of parenchymal-sparing sleeve reconstructions versus radical resections.

### 2.8. Histopathological Diagnosis Workflow

Preoperative biopsy specimens were processed for core H&E-based morphological diagnosis, with immunohistochemical validation and molecular subtyping reserved for post-operative resected specimens; fixation, sectioning, and multi-system correlation details are provided in Supplementary Material, Section S2.

### 2.9. Respiratory Functional Explorations and Pulmonary Reserve Stratification

All patients evaluated at our institution entered a prospective, standardized, and strictly regulated clinical pathway for respiratory functional stratification, in accordance with our established internal department protocol. This institutional care pathway was structured into three sequential tiers with explicit escalation rules to systematically determine parenchymal reserves and maximize perioperative safety.

Tier 1 comprised routine spirometry and body plethysmography, which were performed mandatorily for all 93 screened patients at admission as a baseline institutional screening standard to determine initial forced expiratory volume in 1 s (FEV_1_), forced vital capacity (FVC), and the FEV_1_/FVC ratio. At this initial stage, baseline evaluations identified cases with immediate contraindications or baseline limitations, while the remaining patient population advanced into the structured functional protocol.

Tier 2 escalation involved the measurement of DLCO and the transfer coefficient (KCO) using the single-breath method. Under our institutional protocol, DLCO testing was selectively but automatically triggered by any of the following predefined department criteria: (1) a planned major anatomical resection, including lobectomy, bilobectomy, or pneumonectomy, requiring calculation of expected parenchymal loss; (2) a baseline FEV_1_ below 80% of the predicted value during Tier 1 routine testing; or (3) a significant smoking history exceeding 20 pack-years or subjective dyspnea graded 2 or higher on the modified Medical Research Council scale. For all patients undergoing anatomical resections, the predicted postoperative values (ppoFEV_1_ and ppoDLCO) were rigorously calculated based on the number of functional segments planned for resection, using the segmental lung anatomy formula:

ppoFEV_1_ = preopFEV_1_ × (1 − y/z),

where y represents the number of functional segments planned for resection and z the total functional segments (19 for anatomically healthy lungs) [15,16]; ppoDLCO was derived analogously. A total of 45 patients presented both ppoFEV_1_ and ppoDLCO equal to or greater than 40% of the predicted values and were cleared directly for tailored surgical intervention according to our standard protocol, following formal cardiological clearance. The remaining patients who presented values below the 40% threshold for either ppoFEV_1_ or ppoDLCO, alongside specific cases requiring advanced metabolic characterization, were escalated to the next stage.

Tier 3 escalation involved advanced functional risk stratification via CPET on a cycle ergometer, representing the final mandatory tier of our institutional algorithm. A VO_2_peak below 10 mL/kg/min served as an absolute institutional threshold for severe cardiorespiratory risk. In total, CPET was performed for 28 patients from the screening pool. Within the subgroup of 36 excluded patients, 16 individuals underwent CPET, demonstrating a low mean VO_2_peak of 11.2 mL/kg/min; among them, 14 patients strictly failed to achieve the minimum metabolic compensation threshold of 10 mL/kg/min and were definitively excluded under the category of severe functional respiratory compromise. Within the subgroup of 57 operated patients, 12 individuals required CPET escalation due to borderline ppoFEV_1_ or ppoDLCO values, and all 12 successfully demonstrated satisfactory metabolic compensation with a mean VO_2_peak of 16.5 mL/kg/min, receiving final surgical clearance. Combined with the baseline screening exclusions for advanced oncological stage (*n* = 12), high-risk cardiovascular comorbidities (*n* = 6), and documented patient refusal (*n* = 4), this multi-tiered functional protocol effectively stratified the initial 93 individuals into 36 total exclusions and 57 finalized surgical cases.

### 2.10. Standard Laboratory and Tumor Biomarker Assessment

Standard preoperative laboratory testing (complete blood count, coagulation panel, renal and hepatic function, electrolytes, acute-phase reactants) and airway microbiological screening (sputum and endobronchial aspirate cultures) were performed for all patients under fasting conditions. A panel of serum tumor biomarkers (SCC-Ag, CYFRA 21-1, CEA, proGRP, NSE, and CgA) was also quantified by ELISA at admission and correlated with tumor volume, airway obstruction parameters, and functional decline metrics. Full laboratory and biomarker assay details are provided in Supplementary Material, Section S3.

**Figure 4:**
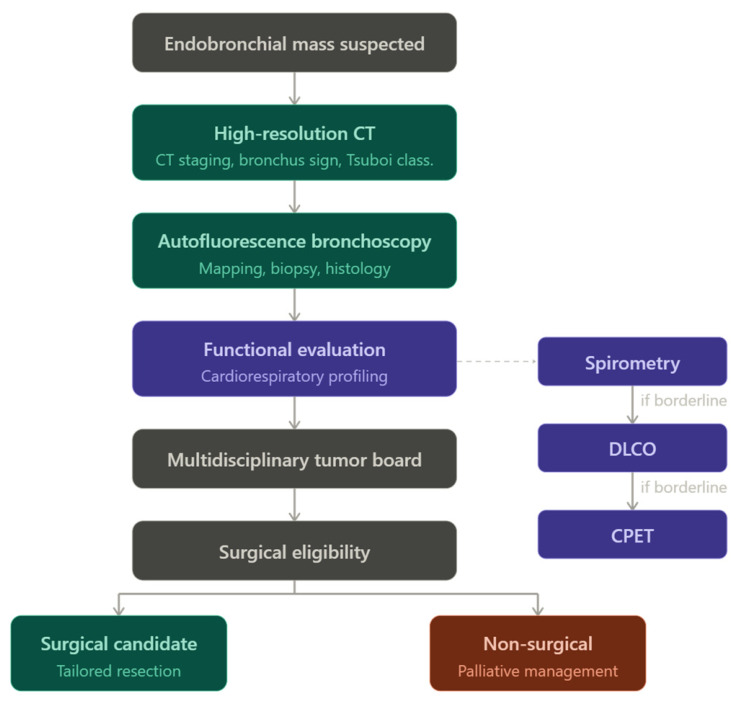
Diagnostic and management algorithm for suspected endobronchial masses.

The institutional diagnostic decision algorithm followed a sequential, multi-system structure: initial locoregional staging via high-resolution CT was followed by flexible AFB for endoscopic mapping and biopsy, integrated with tiered cardiorespiratory functional evaluation (spirometry → DLCO → CPET). All findings were consolidated at a multidisciplinary tumor board review, where anatomical resectability, functional eligibility, and oncological stage were jointly assessed to determine definitive surgical candidacy and intervention type (Figure 4).

### 2.11. Generative AI Statement

During the preparation of this study, generative artificial intelligence tools, specifically Google Gemini and Claude Sonnet 5, were utilized to assist the authors in manuscript refinement. These large language models were employed for advanced English language editing, improving text coherence, ensuring mathematical and statistical consistency between the narrative results and the descriptive tables, and verifying structural compliance with the journal guidelines. These tools were not used to generate, alter, or manipulate raw clinical data, nor did they influence the study design, patient selection, or final clinical interpretations, which remain entirely the responsibility of the authors.

## 3. Results

To provide a transparent overview of the patient selection process, the baseline characteristics and functional parameters of the 36 excluded patients were compared with those of the 57 patients who underwent surgery, with the complete comparative analysis transferred to Supplementary Table S1. The study population comprised 57 patients who underwent primary surgical resection (Table 1). The primary reasons for exclusion from surgical intervention in the 36 patients were, in order of frequency: severe functional respiratory compromise, including a VO_2_peak below 10 mL/kg/min during advanced testing (*n* = 14, 38.9%); advanced or unresectable oncological stage (*n* = 12, 33.3%); high-risk cardiovascular comorbidities, including elevated ThRCRI or ASA IV status (*n* = 6, 16.7%); and documented patient refusal (*n* = 4, 11.1%), consistent with the detailed breakdown provided in Supplementary Table S1.

Regarding the baseline characteristics of the surgical cohort, the clinical and biological analysis revealed a population with a mean age of 61.2 years and a balanced sex distribution, presenting a severity profile dominated by ASA classes II and III. Evaluation of preoperative hematological markers showed stable hemoglobin values, which demonstrated no significant correlations with the main pulmonary functional parameters, yielding a Spearman rho coefficient of 0.148 for DLCO (*p* = 0.2700) and 0.015 for FEV_1_ (*p* = 0.9000). This lack of covariance indicates that anemic status did not act as a confounding factor in the functional selection process. In contrast, major statistical associations were identified between patient age and diffusion capacity, revealing a strong and highly significant inverse correlation (*ρ* = −0.715, *p* < 0.0001). Tumor dimensions showed a positive correlation exclusively with FEV_1_ variability (*ρ* = 0.380, *p* = 0.0035), without exerting a significant impact on DLCO. For full transparency and to provide a comprehensive view of individual patient data, detailed patient-level metrics including surgical procedures, staging, and clinical outcomes are provided in Supplementary Table S2, while the underlying statistical associations between preoperative functional metrics and baseline characteristics are detailed in Supplementary Table S3.

The overall postoperative morbidity rate affected 21 patients (36.84%), with complication rates correlating with both the extent of resection and the completeness of preoperative functional evaluation. Postoperative morbidity was graded using the validated Clavien–Dindo classification system (Table 2), with a detailed stratification of complications by patient-level and event-level provided in Supplementary Table S4. Mechanical and parenchymal-respiratory complications included acute atelectasis in 10 cases (17.54%) and acute respiratory failure—encompassing pneumonia, bronchopneumonia, and ARDS—in 17 cases (29.82%). Prolonged air leaks lasting more than 7 days occurred in 4 patients (7.01%), postoperative hemorrhage was noted in 2 cases (3.50%), and an early bronchial stump fistula developed in 1 patient (1.75%). Cardiovascular morbidity manifested as cardiac arrhythmias in 6 patients (10.52%), acute pulmonary thromboembolism in 2 patients (3.50%), and acute exacerbations of coronary heart disease, such as angina pectoris or acute myocardial infarction, in 3 patients (5.26%). Neurological complications occurred in 2 patients (3.50%).

Surgical outcomes, baseline data, and functional status were stratified according to the specific type of surgical procedure performed. To objectively support the safety profile of parenchymal sparing approaches, a direct comparative analysis was performed between the sleeve resection subgroup (*n* = 17) and patients undergoing major radical resections (*n* = 40; combining lobectomies, bilobectomies, and pneumonectomies); the overall complication rate was significantly lower for parenchymal sparing sleeve resections compared to major lung resections (17.6% vs. 45.0%, *p* = 0.041). Furthermore, when evaluating severe postoperative morbidity, parenchymal sparing techniques demonstrated a superior safety profile, with a major complication rate (Clavien–Dindo Grade III–IV) of only 5.8% (*n* = 1) compared to 20.0% (*n* = 8) documented in the major resection cohort, although this trend did not reach full statistical significance due to sample size constraints (*p* = 0.151). Perioperative mortality was 0.0% for sleeve procedures compared to 7.5% (*n* = 3) for the major resection group (*p* = 0.283), confirming that fatal outcomes were strictly confined to extended parenchymal sacrifices. A direct comparison of outcomes across all four distinct resection types via the multi-group exact test confirmed a significant, stepwise escalation in overall complication rates from parenchymal-sparing sleeve resections (17.6%) to standard lobectomies (40.7%), bilobectomies (50.0%), and radical pneumonectomies (60.0%) (exact multi-group *p* = 0.042). Severe morbidity was also significantly higher in the pneumonectomy group compared to the sleeve group (40.0% vs. 5.8%, *p* = 0.015).

Stratification of postoperative complications by preoperative evaluation pathway identified distinct risk profiles across the three testing pathways within the operated cohort (exact multi-group *p* = 0.038). In the Spirometry-alone pathway (*n* = 14), the complication rate was 21.4% (3 out of 14), consisting entirely of minor respiratory events. In the Spirometry-DLCO pathway (*n* = 23), the complication rate was 52.2% (12 out of 23), with an 8.7% perioperative mortality (2 out of 23). Patients who cleared screening criteria based on these protocols without advanced metabolic evaluation via CPET exhibited a higher rate of cardiac complications, particularly new-onset tachyarrhythmias and acute ischemic events, alongside acute respiratory complications like severe atelectasis and pneumonia. In the Complete Tri-parametric pathway (*n* = 20), which included the highest-risk borderline functional candidates who required cycle ergometer CPET, the complication rate was 30.0% (6 out of 20), with a perioperative mortality rate of 5.0% (1 out of 20). The results of CPET confirmed the utility of the method in risk differentiation for borderline patients; while patients excluded from resection recorded a severely reduced mean exercise performance, with a VO_2_peak of 11.2 mL/kg/min, the cohort of 57 selected and operated patients demonstrated a net superior functional reserve, reaching a mean VO_2_peak of 16.5 mL/kg/min.

Regarding oncological management, all cases were retrospectively restaged and classified according to the newly released 9th edition of the TNM classification for lung cancer. Oncological staging identified Stage II disease in 21 cases (36.8%) and Stage IIIA in 36 cases (63.2%). No Stage I cases were observed, reflecting the central and obstructive nature of these tumors, which typically manifest clinically at more advanced locoregional stages. Direct surgical resection was prioritized over neoadjuvant therapy primarily for central tumors causing high-grade airway obstruction or recurrent post-obstructive pneumonia, to achieve immediate symptom relief and infection control. Histopathological analysis of the definitive specimens confirmed squamous cell carcinoma in 25 cases (43.9%), carcinoid tumors in 17 cases (29.8%), and adenocarcinoma in 15 cases (26.3%). Diagnostic profiles of the surgical cohort were characterized by distinct associations between definitive histopathological tumor types and preoperative serum biomarker levels (Cyfra 21-1, SCC-Ag, NSE, 5-HIAA, CgA, and CEA). These correlations are synthesized in Table 3, which highlights the specific antigenic affinity observed for each pathological subtype.

The overall perioperative mortality rate was 5.26% (*n* = 3). Two fatalities occurred in patients evaluated via spirometry combined with DLCO without CPET: one died of acute myocardial infarction following left upper lobectomy, and the other of pulmonary thromboembolism following left pneumonectomy. The third fatal outcome occurred in a patient who cleared the complete tri-parametric protocol (spirometry, DLCO, and CPET) and subsequently died of severe intractable ARDS following radical right pneumonectomy. All three fatal outcomes occurred exclusively following major resections.

## 4. Discussion

Our study evaluates an integrated preoperative protocol for selecting candidates between parenchymal-sparing sleeve resections and major anatomical lung resections in central endobronchial neoplasms. The operative cohort (*n* = 57) demonstrated significant postoperative morbidity and a clear perioperative mortality rate [17,18,19]. These findings align with our institutional database of 1369 NSCLC patients, confirming that tumor stage and resection completeness remain the primary independent determinants of survival [20].

Parenchymal-conserving airway surgery yields favorable functional outcomes compared to radical resections [21]. Reconstructive planning relies on chest CT and autofluorescence imaging (AFI) bronchoscopy to identify objective resectability markers, such as two tumor-free cartilaginous rings [9,10]. AFI bronchoscopy improves diagnostic yield by detecting mucosal alterations invisible under white light [22], although small forceps biopsy volumes often limit preoperative immunohistochemical profiling.

Serological and urinary tumor biomarker screening, encompassing Cyfra 21-1, NSE, CEA, SCC-Ag, CgA, and urinary 5-HIAA, represents a complementary tool in the thoracic oncology workup [23,24,25,26]. These markers lack absolute organ specificity and must be interpreted within the full clinical context; for instance, marked Cyfra 21-1 elevation occurs in advanced urothelial carcinomas [27]. Within our neuroendocrine sub-cohort, ectopic hormone expressions underlying Cushing and carcinoid syndromes resolved following complete surgical resection [28]. Although preoperative biomarker concentrations showed no significant correlation with perioperative morbidity or mortality (*p* > 0.05), they demonstrated strong cohort-specific affinities, including SCC-Ag for squamous cell carcinoma (r = 0.829, *p* < 0.001), CEA for adenocarcinoma, and CgA for carcinoid biology. Baseline elevations of Cyfra 21-1 and CEA track with reduced disease-free survival and increased recurrence, positioning them as valuable adjuncts for long-term disease monitoring.

Contemporary literature increasingly emphasizes the role of systemic inflammatory and immune biomarkers in predicting short-term surgical outcomes. Baseline inflammatory metrics like C-reactive protein (CRP) and absolute eosinophil counts (EOS) have emerged as indicators of perioperative vulnerability, with recent multicenter data demonstrating that baseline eosinophil levels are significantly associated with complication trajectories after pulmonary lobectomies [29]. Our cohort documented preoperative leukocytosis in 21.1% of cases, reflecting an underlying inflammatory state secondary to central neoplastic processes and recurrent post-obstructive pneumonia. Although specific sub-analyses for isolated baseline eosinophils or high-sensitivity CRP were not systematically tracked as independent predictors in our initial protocol, integrating these inflammatory axes represents a crucial direction for refining future risk stratification pathways.

Preoperative risk stratification relied on multimodal physiological evaluation. Baseline testing primarily showed mild-to-moderate ventilatory dysfunction, where the interplay between preoperative FEV1, ppoFEV1, and hemodynamic status guided operative safety margins [16,30], applying an absolute FEV1 threshold of 1 L for borderline operability and 800 mL for functional inoperability. CPET remains the reference standard for evaluating candidates, providing a dynamic assessment of cardiopulmonary, metabolic, and musculoskeletal reserves under exertional stress [16,31,32], where VO_2_peak acts as a strong independent predictor of postoperative respiratory morbidity [33]. Values below 10 mL/kg/min represented a functional contraindication, while values above 20 mL/kg/min permitted pneumonectomy within a low-risk classification. The ThRCRI complemented this paradigm by identifying borderline candidates requiring additional invasive cardiovascular evaluation [5], placing all 57 operated patients within low-to-moderate risk classes A and B. For functionally ineligible patients, endobronchial alternatives like argon plasma coagulation, diode laser, electrocautery debulking, and cryoresection provided local tumor control and symptom palliation [34,35,36].

Crucially, patients undergoing radical right pneumonectomy remained at risk for severe perioperative complications even after complete tri-parametric functional clearance. The anatomical and hemodynamic magnitude of this intervention introduces acute pulmonary vascular resistance shifts and sudden volumetric changes that transcend standard cardiorespiratory scores, rendering parenchymal sacrifice an independent risk modifier. Conversely, the zero mortality and favorable safety profile in the sleeve resection subgroup reflect inherent selection bias, as these patients represent a carefully selected subset where lung preservation was prioritized. Nonetheless, the two fatal cardiovascular events (an acute myocardial infarction following lobectomy and a pulmonary thromboembolism following pneumonectomy) occurred exclusively in the resting functional pathways without CPET, underscoring latent vascular liabilities that evade resting tests and reinforcing the value of complete tri-parametric evaluation.

The higher rate of cardiac complications in spirometry-only or spirometry-DLCO pathways may be confounded by non-random diagnostic allocation, where low-risk classification based on unremarkable resting tests underestimated occult disease, whereas CPET was preferentially ordered for borderline candidates, resulting in closer perioperative surveillance and targeted medical optimization. Documented complications followed two distinct pathophysiological patterns: technical trauma (early bronchial stump fistulas and prolonged air leaks) and functional underestimation, where resting parameters masked latent microvascular or alveolar-capillary frailty during one-lung ventilation. Because standard algorithms fail to capture preoperative vascular status, our postoperative neurological (*n* = 2) and acute coronary (*n* = 3) events indicate silent cerebrovascular and coronary atherosclerotic disease undetected by ThRCRI-based stratification. To address this, we suggest the systematic incorporation of carotid artery Doppler ultrasound and selective CT angiography of the carotid and coronary territories into the preoperative protocol for candidates with a high atherosclerotic burden.

Refining these multi-parametric selection criteria supports safer parenchymal preservation, reducing the necessity for radical pneumonectomies. While the cohort exhibits histopathological heterogeneity typical of central endobronchial neoplasms, the limited number of mucoepidermoid carcinoma cases (*n* = 4) precludes meaningful clinical interpretation and remains highly susceptible to small-sample constraints. The complete absence of Stage I cases underscores that central obstructive tumors typically produce symptoms only at advanced locoregional stages. Furthermore, institutional morphological analyses show that specific stromal desmoplasia, tumor budding, and keratinization patterns independently influence survival in resected squamous cell carcinoma [37]. Although long-term survival trajectories vary by lineage, our algorithm prioritized immediate anatomical resectability and perioperative cardiorespiratory risk over histology-specific prognosis. Given an institutional median overall survival of 49 months [20], these multi-parametric criteria remain highly applicable regardless of long-term outcomes, aligning with evolving risk stratification guidelines for complex thoracic reconstructions [38].

### Study Limitations

Several limitations of this study must be acknowledged. Its retrospective, single-center design restricts external generalizability and direct causal inferences. The small sample size of 57 surgical patients and the concentration of adverse events within major resection subgroups precluded formal multivariate logistic regression modeling. Additionally, long-term oncological outcomes were not tracked, CPET was not applied uniformly to the entire population, and the retrospective database lacked an itemized comorbidity registry, approximating overall burden through composite indices like the ASA score and ThRCRI.

Based on these findings, we propose that a tri-parametric functional evaluation combining spirometry, DLCO, and CPET should be considered for major resection candidates to optimize risk stratification. Right-sided radical pneumonectomy must be recognized as an independent risk modifier during multidisciplinary tumor board reviews, irrespective of functional clearance. Finally, for patients deemed functionally ineligible for major surgery, endobronchial interventional alternatives like argon plasma coagulation or cryoresection should be systematically offered as a structured palliative pathway to maintain local tumor control.

## 5. Conclusions

In conclusion, defining precise selection criteria for parenchymal-sparing sleeve resections in patients with central endobronchial tumors remains a technically and clinically evolving challenge in thoracic oncology. Our single-center retrospective findings offer exploratory observations demonstrating that perioperative risk stratification and complication predictability are enhanced when utilizing a complete tri-parametric pathway combining spirometry, DLCO, and CPET.

However, these data should be interpreted strictly within the limitations of an exploratory cohort study rather than as a definitive validation of a predictive algorithm. The anatomical and hemodynamic magnitude of major surgical interventions—particularly radical pneumonectomy—constitutes an independent risk modifier that can exceed the predictive capacity of preoperative functional testing alone. Therefore, large-scale prospective multi-center trials are needed to further explore the generalizability of integrated respiratory, cardiovascular, and biomarker-based screening models, incorporating recently updated staging systems, including the ninth edition of the TNM classification.

## Figures and Tables

**Table 1:** Baseline Clinical, Functional, and Oncological Characteristics of the Operated Cohort (*n* = 57).

Parameter	Value
Demographics	
Age (years, mean ± SD)	61.2 ± 6.8
Sex (Male/Female)	38/19
Comorbidity and Risk Stratification (composite indices)	
ASA Physical Status Class II	28 (49.1%)
ASA Physical Status Class III	26 (45.6%)
ASA Physical Status Class IV	3 (5.3%)
ThRCRI Risk Class A/B (low-to-moderate)	57 (100.0%)
Type of Operation	
Sleeve Resection	17 (29.8%)
Standard Lobectomy	27 (47.4%)
Bilobectomy	8 (14.0%)
Pneumonectomy	5 (8.8%)
Tumor Histology	
Squamous Cell Carcinoma	25 (43.9%)
Adenocarcinoma	15 (26.3%)
Carcinoid Tumor	17 (29.8%)
Tumor Location	
Central	25 (43.9%)
Peripheral	32 (56.1%)

Comorbidity burden is captured via the ASA class and ThRCRI composite indices rather than an itemized diagnosis list (see Limitations); hematological values reflect preoperative peripheral blood work, detailed further in Section 3. Note: ASA (American Society of Anesthesiologists) physical status class and the Thoracic Revised Cardiac Risk Index (ThRCRI) are reported as the validated composite indices used in this cohort to summarize overall comorbidity and perioperative risk burden; an itemized registry of individual comorbid diagnoses (e.g., diabetes, hypertension, chronic obstructive pulmonary disease) was not available in our retrospective institutional database (see Limitations). Anemia, leukocytosis, and thrombocytosis are preoperative peripheral blood abnormalities detailed further in Section 3 (Results).

**Table 2:** Postoperative Outcomes and Complication Rates Stratified by Surgical Procedure (*n* = 57).

Parameter	Sleeve Resection (*n* = 17)	Standard Lobectomy (*n* = 27)	Bilobectomy (*n* = 8)	Pneumonectomy (*n* = 5)
Overall Complication Rate	17.6% (*n* = 3)	40.7% (*n* = 11)	50.0% (*n* = 4)	60.0% (*n* = 3)
95% Confidence Interval	3.8% to 43.4%	22.4% to 61.2%	15.7% to 84.3%	14.7% to 94.7%
Clavien–Dindo Grade I–II	2 (11.7%)	7 (25.9%)	2 (25.0%)	1 (20.0%)
Clavien–Dindo Grade III–IV	1 (5.8%)	4 (14.8%)	2 (25.0%)	2 (40.0%)
Perioperative Mortality (Grade V)	0 (0.0%)	1 (3.7%)	0 (0.0%)	2 (40.0%)

**Table 3:** Correlation between Histopathological Tumor Types and Preoperative Biomarkers.

Histopathological Type	Primary Associated Marker	Correlation Coefficient (r)	*p*-Value
Squamous Cell Carcinoma	SCC-Ag	0.829	<0.001
Adenocarcinoma	CEA	0.426	0.0009
Carcinoid	CgA	0.356	0.0100

## Data Availability

The original contributions presented in this study are included in the article/Supplementary Material. Further inquiries can be directed to the corresponding authors.

## Supplementary Materials

The following supporting information can be downloaded at: https://www.mdpi.com/article/10.3390/jcm15145605/s1, Section S1: Computed Tomography Acquisition and Classification Protocol; Section S2: Detailed Endobronchial Sampling and Histopathological Processing Protocol Tissue Fixation and Processing; Section S3: Standard Preoperative Laboratory and Tumor Biomarker Assessment Protocol; Table S1: Comparative analysis of clinical, functional, and oncological characteristics between excluded and operated patients; Table S2: Detailed Patient Characteristics and Outcomes by Surgical Procedure (*n* = 57); Table S3: Consolidated Spearman Rank Correlation Matrix of Preoperative Functional Metrics (DLCO and FEV1) and Baseline Clinical-Biological Characteristics (*n* = 57); Table S4: Stratification of Postoperative Complications by Patient-Level and Event-Level.

## Abbreviations

The following abbreviations are used in this manuscript:

5-HIAA	5-Hydroxyindoleacetic Acid
AFB	Autofluorescence Bronchoscopy
AFI	Autofluorescence Imaging
AMI	Acute Myocardial Infarction
APC	APC: Argon Plasma Coagulation
ARDS	Acute Respiratory Distress Syndrome
ASA	American Society of Anesthesiologists
CBC	Complete Blood Count
CEA	Carcinoembryonic Antigen
CgA	Chromogranin A
CPET	Cardiopulmonary Exercise Testing
CT	Computed Tomography
CT-BS	CT Bronchus Sign
Cyfra 21-1	Cytokeratin Fragment 19 Antigen
DLCO	Diffusing Capacity of the Lung for Carbon Monoxide
ELISA	Enzyme-Linked Immunosorbent Assay
ESMO	European Society for Medical Oncology
FEV_1_	Forced Expiratory Volume in 1 Second
FFPE	Formalin-Fixed Paraffin-Embedded
FVC	Forced Vital Capacity
GOLD	Global Initiative for Chronic Obstructive Lung Disease
IHC	Immunohistochemistry
INR	International Normalized Ratio
KCO	DLCO per Unit of Alveolar Volume
NCCN	National Comprehensive Cancer Network
NSE	Neuron-Specific Enolase
NSCLC	Non-Small Cell Lung Cancer
ppoDLCO	Predicted Postoperative DLCO
ppoFEV_1_	Predicted Postoperative FEV_1_
ProGRP	Progastrin-Releasing Peptide
RV	Residual Volume
SCC-Ag	Squamous Cell Carcinoma Antigen
SD	Standard Deviation
ThRCRI	Thoracic Revised Cardiac Risk Index
TIA	Transient Ischemic Attack
TNM	Tumor, Node, Metastasis
VO_2_peak	Peak Oxygen Consumption
