# Supplementary material for: Functional Stratification and Postoperative Outcomes in Endobronchial Tumor Surgery: A Dedicated Center Experience
Source: J Clin Med. 2026 Jul 17;15(14):5605. doi: 10.3390/jcm15145605 (PMC13412668; doi:10.3390/jcm15145605)
Supplement: Supplementary file 1 [file jcm-15-05605-s001.zip › jcm-4394091-supplementary.pdf]

Supplementary Table S1. Comparative analysis of clinical, functional, and oncological characteristics between excluded and operated patients.

Parameter	Excluded Patients (n = 36)	Operated Patients (n = 57)
Age (years, mean ± SD)	66.4 ± 7.1	61.2 ± 6.8
**Sex (n, %)**		
Male	24 (66.7%)	38 (66.7%)
Female	12 (33.3%)	19 (33.3%)
**ASA Score (n, %)**		
Class II	10 (27.8%)	28 (49.1%)
Class III	22 (61.1%)	26 (45.6%)
Class IV	4 (11.1%)	3 (5.3%)
FEV1 (% predicted, mean ± SD)	52.3 ± 8.4	78.4 ± 11.2
DLCO (% predicted, mean ± SD)	48.1 ± 7.9	74.2 ± 10.5
CPET VO2peak (mL/kg/min, mean)	11.2 (n = 16 tested)	16.5 (n = 12 tested)
**Tumor Stage (n, %)**		
Stage I	2 (5.6%)	0 (0.0%)
Stage II	5 (13.9%)	21 (36.8)
Stage III	18 (50.0%)	36 (63.2%)
Stage IV	11 (30.6%)	0 (0.0%)
**Tumor Location (n, %)**		
Central	21 (58.3%)	25 (43.9%)
Peripheral	15 (41.7%)	32 (56.1%)
**Primary Reason for Exclusion (n, %)**		
Respiratory Insufficiency	14 (38.9%)	Not applicable
Advanced Oncological Stage	12 (33.3%)	Not applicable
Severe Cardiovascular Risk	6 (16.7%)	Not applicable
Patient Refusal	4 (11.1%)	Not applicable

Section S1: Computed Tomography Acquisition and Classification Protocol

High-resolution thoracic CT scans were performed for all patients to evaluate tumor dimensions, structural characteristics, and the precise anatomical boundaries of the lesions. The extent of airway involvement and tumor localization were systematically categorized according to the Tsuboi classification system for central lung tumors. Radiographic signs, including peripheral atelectasis, secondary pneumonitis, and contiguity with adjacent vascular structures, were documented to aid the multidisciplinary tumor board in assessing surgical resectability.

Section S2: Detailed Endobronchial Sampling and Histopathological Processing Protocol Tissue Fixation and Processing:

Biopsy specimens obtained via serial forceps biopsies during flexible bronchoscopy were fixed in 10% buffered formalin and processed using standard formalin-fixed paraffin-embedded (FFPE) techniques. The fixation period was maintained between 12 and 24 hours to ensure structural preservation before the specimens underwent automated dehydration through graded ethanol solutions, clearing in xylene, and final infiltration with molten paraffin wax. Sectioning and Staining:

Paraffin blocks were sectioned to generate serial histological sections, which were subsequently stained with hematoxylin and eosin (H&E) for comprehensive light microscopy evaluation. The diagnostic workflow was optimized to prioritize core morphological characterization and architectural grading due to the limited volume of tissue retrieved. Routine preoperative immunohistochemical (IHC) profiling was omitted on these initial small biopsy specimens, reserving definitive IHC validation and extensive molecular subtyping for the post-operative resected surgical specimens. Multi-System Correlation Protocol Framework:

To supplement pre-resection tissue biopsies, the histopathological protocol integrated baseline tissue findings with peripheral laboratory parameters, including serum tumor markers, and systemic functional assessments. This multi-system correlation protocol was implemented to assess tumor biological behavior by correlating metabolic, cellular, and functional parameters with perioperative outcomes and post-resection clinical safety.

Section S3: Standard Preoperative Laboratory and Tumor Biomarker Assessment Protocol

Peripheral venous blood samples and biological specimens were collected from all patients upon hospital admission under fasting conditions. The preoperative laboratory workup comprised a comprehensive multi-system homeostatic evaluation designed to assess perioperative physiological status. This panel included a complete blood count (CBC) with automated differential leukocyte profiling, full coagulation panels (including prothrombin time, INR, and aPTT) to verify adequate surgical hemostasis, and standard automated metabolic profiles assessing renal function, hepatic function, serum electrolytes, and acute-phase reactants. Additionally, targeted microbiological screening, including sputum cultures and

endobronchial aspirate bacteriological testing, was conducted to identify airway colonization or active infections.

To assist in baseline oncological characterization, supplement histopathological profiling, and establish a comparative postoperative monitoring benchmark, a panel of serum tumor biomarkers was analyzed for all enrolled patients upon admission. Quantitative enzyme-linked immunosorbent assays (ELISAs) were used to determine the baseline peripheral concentrations of squamous cell carcinoma antigen (SCC-Ag), cytokeratin fragment 19 antigen (CYFRA 21-1), carcinoembryonic antigen (CEA), progastrin-releasing peptide (proGRP), neuron-specific enolase (NSE), and chromogranin A (CgA). These concentrations were analyzed as continuous variables and correlated with tumor volume, anatomical airway obstruction parameters, and functional decline metrics.

Supplementary Table S2. Detailed Patient Characteristics and Outcomes by Surgical Procedure (n = 57)

ID	Procedure	Age	ASA	FEV1	ppoFEV1	DLCO	ppoDLCO	CPET VO2	ThRCRI	Stage	Histology	Complications	Mortality
**1**	Sleeve	58	II	93	86	86	80	N/A	2	II	Carcinoid	None	No
**2**	Sleeve	58	II	89	82	84	78	19	1	II	Carcinoid	None	No
**3**	Sleeve	58	II	94	87	88	82	N/A	1	II	Carcinoid	None	No
**4**	Sleeve	59	II	98	91	90	84	N/A	1	II	Carcinoid	None	No
**5**	Sleeve	53	II	87	80	83	77	N/A	1	III	Carcinoid	Minor Resp	No
**6**	Sleeve	45	II	88	81	85	79	N/A	1	III	Carcinoid	None	No
**7**	Sleeve	47	II	99	92	95	89	22	1	II	Carcinoid	None	No
**8**	Sleeve	46	II	95	88	91	85	N/A	1	II	Carcinoid	Major	No
**9**	Sleeve	54	II	89	82	82	76	21	1	II	Carcinoid	None	No
**10**	Sleeve	52	II	93	86	89	83	N/A	1	II	Carcinoid	None	No
**11**	Sleeve	58	II	96	89	95	89	N/A	1	II	Carcinoid	Minor Resp	No
**12**	Sleeve	49	II	87	80	82	76	N/A	1	II	Carcinoid	None	No
**13**	Sleeve	59	II	99	92	96	90	18	1	III	Carcinoid	None	No
**14**	Sleeve	43	II	95	88	93	87	16	1	II	Carcinoid	None	No
**15**	Sleeve	49	II	91	84	86	80	N/A	1	II	Carcinoid	None	No
**16**	Sleeve	59	II	87	80	86	80	19	1	II	Carcinoid	None	No
**17**	Sleeve	54	II	88	81	83	77	12	1	II	Carcinoid	None	No
**18**	Lobectomy	58	III	81	71	80	71	N/A	2	II	Adeno	None	No
**19**	Lobectomy	68	III	80	70	77	68	13	3	II	Squamous	None	No
**20**	Lobectomy	65	III	69	59	62	53	N/A	2	III	Adeno	None	No
**21**	Lobectomy	70	III	74	64	70	61	17	2	III	Squamous	Minor Resp	No
**22**	Lobectomy	56	II	69	59	67	58	N/A	1	III	Adeno	None	No
**23**	Lobectomy	68	II	85	75	79	70	N/A	2	III	Adeno	Major	Yes
**24**	Lobectomy	64	III	80	70	77	68	N/A	3	III	Adeno	None	No
**25**	Lobectomy	60	II	68	58	64	55	N/A	1	III	Squamous	None	No
**26**	Lobectomy	66	III	86	76	81	72	N/A	3	III	Adeno	None	No
**27**	Lobectomy	70	III	71	61	63	54	N/A	3	III	Squamous	None	No
**28**	Lobectomy	58	III	74	64	70	61	19	3	III	Adeno	Minor Resp	No
**29**	Lobectomy	67	III	85	75	84	75	N/A	2	II	Squamous	None	No
**30**	Lobectomy	70	II	68	58	65	56	N/A	1	III	Adeno	None	No
**31**	Lobectomy	60	III	86	76	82	73	N/A	2	II	Squamous	Minor Resp	No
**32**	Lobectomy	64	II	85	75	81	72	N/A	2	III	Adeno	Minor Resp	No
**33**	Lobectomy	62	III	79	69	73	64	19	2	III	Adeno	None	No

**34**	Lobectomy	70	II	68	58	65	56	15	1	III	Adeno	Major	No
**35**	Lobectomy	58	III	74	64	71	62	12	3	III	Squamous	None	No
**36**	Lobectomy	62	II	69	59	61	52	N/A	1	II	Adeno	None	No
**37**	Lobectomy	69	III	84	74	79	70	N/A	2	III	Squamous	Minor Resp	No
**38**	Lobectomy	59	III	71	61	63	54	11	3	III	Squamous	None	No
**39**	Lobectomy	64	II	77	67	72	63	N/A	1	III	Squamous	Minor Resp	No
**40**	Lobectomy	65	II	80	70	76	67	N/A	1	III	Adeno	None	No
**41**	Lobectomy	68	III	71	61	65	56	N/A	2	III	Squamous	Major	No
**42**	Lobectomy	64	II	84	74	79	70	11	1	III	Squamous	Minor Resp	No
**43**	Lobectomy	61	II	71	61	68	59	N/A	1	III	Squamous	Major	No
**44**	Lobectomy	66	III	85	75	79	70	N/A	3	II	Squamous	None	No
**45**	Bilobectomy	64	III	64	54	59	50	N/A	2	III	Adeno	None	No
**46**	Bilobectomy	64	III	69	59	67	58	22	2	III	Squamous	Minor Resp	No
**47**	Bilobectomy	64	III	73	63	71	62	12	2	III	Squamous	None	No
**48**	Bilobectomy	67	III	71	61	69	60	17	3	III	Squamous	Major	No
**49**	Bilobectomy	64	IV	62	52	59	50	N/A	3	III	Squamous	None	No
**50**	Bilobectomy	65	III	61	51	59	50	N/A	2	II	Squamous	Major	No
**51**	Bilobectomy	69	III	70	60	68	59	N/A	3	III	Adeno	Minor Resp	No
**52**	Bilobectomy	68	III	70	60	64	55	16	2	III	Squamous	None	No
**53**	Pneumonectomy	65	IV	59	49	56	47	N/A	3	III	Squamous	Major	Yes
**54**	Pneumonectomy	68	IV	60	50	58	49	N/A	3	III	Squamous	Major	Yes
**55**	Pneumonectomy	64	III	56	46	52	43	N/A	2	III	Squamous	Minor Resp	No
**56**	Pneumonectomy	63	III	56	46	49	40	N/A	2	III	Squamous	None	No
**57**	Pneumonectomy	70	III	56	46	51	42	19	2	III	Squamous	None	No

Note: Complication categories (None / Minor Resp / Major) correspond to the absence of complications, Clavien-Dindo Grade I-II, and Clavien-Dindo Grade III-IV respectively (see Supplementary Table 2 for the event-level breakdown by organ system). Aggregate values in this table are consistent with Table 1 (baseline characteristics), Table 2 (postoperative outcomes by procedure), and Supplementary Table 3 (excluded vs. operated comparison).

Supplementary Table S3. Consolidated Spearman Rank Correlation Matrix of Preoperative Functional Metrics (DLCO and FEV_1) and Baseline Clinical-Biological Characteristics (n = 57).

**Patient Characteristic / Variable**	**Preoperative DLCO Variance (Spearman Coefficient ϱ / p-value)**	**Preoperative FEV_1 Variance (Spearman Coefficient ϱ / p-value)**
Age (Years)	–0.715 / < 0.0001	0.071 / 0.5900
Height (cm)	0.269 / 0.0400	0.583 / < 0.0001
ASA Physical Status Score	–0.548 / < 0.0001	0.170 / 0.2000
Tumor Dimensions (mm)	0.021 / 0.8800	0.380 / 0.0035
Hb Value (g/dL)	0.148 / 0.2700	0.015 / 0.9000
Dyspnea	0.029 / 0.8300	0.179 / 0.1800
Weight Loss	0.046 / 0.7300	0.126 / 0.3500
Hemoptysis	*N/A*	*N/A*
Chest Pain	*N/A*	*N/A*
Cough	*N/A*	*N/A*

Note: **N/A**: Not Applicable. The Spearman rank correlation coefficient could not be mathematically calculated because these clinical symptoms acted as constants with zero or near-zero variance within the definitive surgical cohort (n = 57). Specifically, persistent cough and localized chest pain were present in 100.0% of patients, and hemoptysis was present in 94.7% of patients, preventing covariance calculation due to a zero denominator.

Supplementary Table S4: Stratification of Postoperative Complications by Patient-Level and Event-Level

**Metric Description**	**Count / Value**	**Percentage of Cohort (n=57)**
Patients with at least one complication (Patient-level)	21	36.8%
Total number of adverse events (Event-level)	29	-
Minor Complications (Clavien-Dindo Grade I-II)	19	-
Major Complications (Clavien-Dindo Grade III-IV)	10	-

**Event Type**	**Clavien-Dindo Grade I-II (Minor)**	**Clavien-Dindo Grade III-IV (Major)**	**Total Events**
**Respiratory (e.g., Pneumonia, Air leak)**	10	5	15
**Cardiac (e.g., Arrhythmia)**	5	1	6
**Infectious (e.g., Empyema)**	2	2	4
**Other (e.g., Renal, Neurological)**	2	2	4
**Total**	19	10	29

**Clavien-Dindo Grade**	**Number of Events**
**Grade I**	8
**Grade II**	11
**Grade III**	7
**Grade IV**	3
**Grade V (Mortality)**	3

Note: The total of 3 deaths is included in the event count for full clinical transparency.
